# Identifying term relations cross different gene ontology categories

**DOI:** 10.1186/s12859-017-1959-3

**Published:** 2017-12-28

**Authors:** Jiajie Peng, Honggang Wang, Junya Lu, Weiwei Hui, Yadong Wang, Xuequn Shang

**Affiliations:** 1 School of Computer Science, Northwestern Polytechnical University, Xi’an, China; 2 School of Computer Science and Technology, Harbin Institute of Technology, Harbin, China

**Keywords:** Gene Ontology, Term similarity, Cross categories

## Abstract

**Background:**

The Gene Ontology (GO) is a community-based bioinformatics resource that employs ontologies to represent biological knowledge and describes information about gene and gene product function. GO includes three independent categories: molecular function, biological process and cellular component. For better biological reasoning, identifying the biological relationships between terms in different categories are important.

However, the existing measurements to calculate similarity between terms in different categories are either developed by using the GO data only or only take part of combined gene co-function network information.

**Results:**

We propose an iterative ranking-based method called *C* *r* *o* *G* *O*2 to measure the cross-categories GO term similarities by incorporating level information of GO terms with both direct and indirect interactions in the gene co-function network.

**Conclusions:**

The evaluation test shows that *C* *r* *o* *G* *O*2 performs better than the existing methods. A genome-specific term association network for yeast is also generated by connecting terms with the high confidence score. The linkages in the term association network could be supported by the literature. Given a gene set, the related terms identified by using the association network have overlap with the related terms identified by GO enrichment analysis.

## Background

The Gene Ontology (GO) is a community-based bioinformatics resource that employs ontologies to represent biological knowledge and describes information about gene and gene product function [1]. It is widely used to infer functional information for gene products, such as gene function enrichment [2], protein function prediction [3, 4], disease association analysis [5–7]. GO contains three key categories: cellular component (CC; where gene products are active), molecular function (MF; the biological function of gene or gene product) and biological process (BP; pathways or larger processes that multiple gene products involved in). Comparing the similarity between GO terms is an important basic for the GO-based application. The methods of measuring term similarities have been extensively studied in last decade [8–19]. However, most of existing methods focus on measuring the similarity in the same GO category and cannot calculate the semantic similarities between GO terms belonging to different GO categories.

Although GO is originally constructed as three independent categories, identifying their biological relationships may be helpful to understand the biological mechanism and infer gene function [20]. Furthermore, identifying relationships between terms in different categories may provide evidence for biological reasoning and hypotheses. For example, anaphase-promoting complex plays an important role in anaphase inhibitory protein degradation and mitotic cyclins, which can be revealed by discovering the relationship between MF term “anaphase-promoting complex binding” and BP term “activation of anaphase-promoting complex activity involved in meiotic cell cycle” [21].

Several methods are proposed to calculate the similarities between terms across GO categories. Let *t*_1_ and *t*_2_ be two terms belonging to two different GO categories. Association rule mining (ASR), which is a well-known data mining algorithm, was used to calculate the similarity of *t*_1_ and *t*_2_, labeled as *S* *i* *m*_*ASR*_(*t*_1_,*t*_2_) [22, 23]. By combining the ASR approach and text mining-based method, Myhre et al. generated a ready-for-use cross-category GO structure. The limitation of the ASR-based approach is that “shallow annotation” problem is ignored [24]. Specifically, let *t*_1_ and *t*_2_ be two terms in different categories *C*_1_ and *C*_2_. If both *t*_1_ and *t*_2_ are high-level terms that are near to the root terms of *C*_1_ and *C*_2_, the similarity between *t*_1_ and *t*_2_ may be high no matter whether *t*_1_ and *t*_2_ are biologically related. The reason is that the high-level terms may annotate almost all genes involved in a GO category after propagation [25]. Consequently, term pairs at high levels can have high similarity, which may not reflect the biological relationship between the terms.

To solve the “shallow annotation” problem, a Vector Space Model (VSM)-based approach was developed by Bodenreidar et al.. This method takes the semantic information of genes into account to avoid “shallow annotation” problem. VSM is a classical method, which is widely used to calculate the similarities between documents that can be represented as vectors [23]. Specifically, each term is considered as a vector, which length is the same as all the genes involved in GO. Each element in a vector is a binary value. If there is association between a term and a gene, the binary value is 1, otherwise 0 [26]. The similarity of *t*_1_ and *t*_2_ in different categories can be measured with weighted cosine similarity. The VSM-based approach is based on the interaction of the gene sets annotated by *t*_1_ and *t*_2_. Therefore, the result heavily relies on the quality and coverage of G annotation data. Unfortunately, the gene annotations are far from complete currently [27], which may lead to inaccurate term similarity scores.

To avoid the data availability problem, inspiring from existing integration methods, a novel method CroGO was proposed to calculate the similarity between two GO terms in different categories in our previous work [21]. CroGo incorporate gene co-function network data and gene ontology data to calculate the cross-categories GO term similarities. The experiment result shows that CroGO outperforms the aforementioned methods. However, only part of the information in gene co-function network was used by CroGO, since it only took the direct link in the network into account. Other than the directly connected gene pairs, the indirect gene-gene interactions contained in the gene co-function network should also be considered.

In this paper, we developed a novel approach, *C* *r* *o* *G* *O*2, to measure the cross-categories GO term similarities by incorporating both direct and indirect interactions in the gene co-function network. Comparing with the existing approaches, *C* *r* *o* *G* *O*2 has the following advantages:

- Comparing with the state-of-art methods, *C* *r* *o* *G* *O*2 performs better than existing methods by taking the global interactions in the gene co-functional network into account. It proves that gene co-functional network could be a good complement to GO for cross-categories term similarity calculation.
- A novel iterative ranking-based method is developed to measure the relationship between two gene sets based on the gene co-functional network.
- A cross-categories term association network was constructed by selecting the term-pairs with high similarity score calculated by *C* *r* *o* *G* *O*2. Applying *C* *r* *o* *G* *O*2 to identify the highly related terms between BP and MF category has discovered term pairs with solid supports from literature.

## Methods

**Fig. 1:**
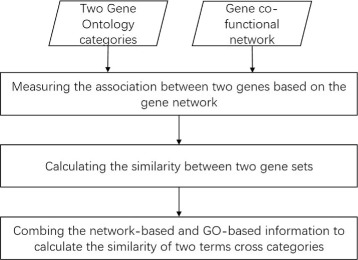
The workflow of *C* *r* *o* *G* *O*2

We proposes *C* *r* *o* *G* *O*2 to measure the relationships between genes based on the global feature of a gene network and then measure the similarity between GO terms in different categories. To measure the similarity of *t*_1_ and *t*_2_ in different categories, *C* *r* *o* *G* *O*2 consists of three steps. First, it measures the interaction between genes based on the gene network. Second, it calculates the similarity between two gene sets annotated by *t*_1_ and *t*_2_ based on gene-gene associations from last step. Third, it combines the network-based gene set similarities and the level information of *t*_1_ and *t*_2_ in GO to calculate the similarity between *t*_1_ and *t*_2_. The diagram of the whole process of *C* *r* *o* *G* *O*2 is shown in Fig. 1.

### Step 1. measuring the network-based association between two genes

In this step, we use both the direct and indirect interactions between genes in the gene co-functional network to measure the association between two genes. A gene network includes not only the direct interaction between genes but also the global view of associations among genes, which are not connected directly. In this step, we adopted the iterative ranking (IR) [28] algorithm to measure the association between two genes. The basic idea is that the

**Fig. 2:**
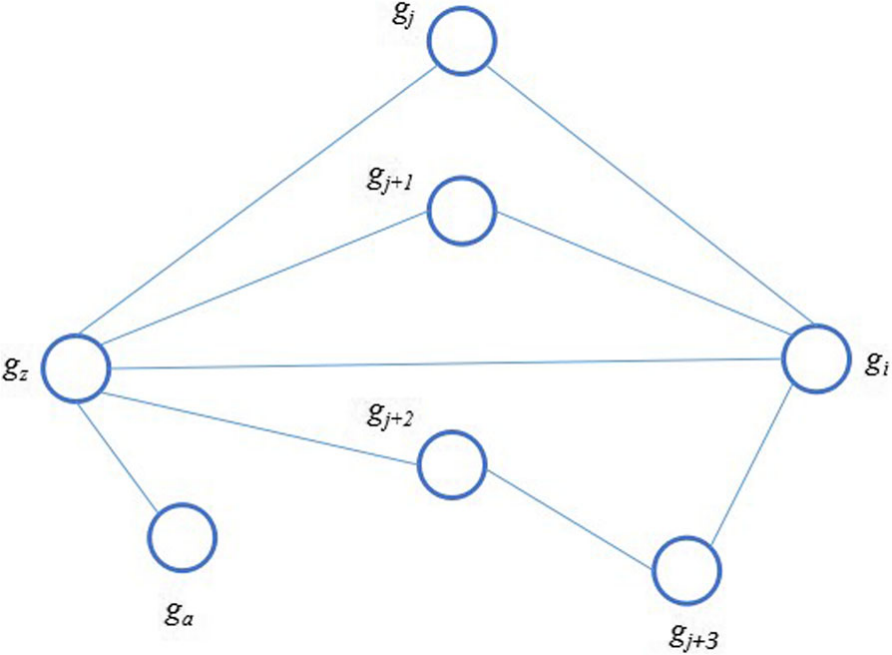
Illustration example for iterative ranking based association score. The nodes and edges represent genes and their interactions respectively

Figure 2 is an illustration example of our basic idea. Given a gene co-functional network *G*(*V*,*E*), the association score between gene *g*_*z*_ and *g*_*i*_ is determined by two types of information: the direct link between *g*_*z*_ and *g*_*i*_, (*g*_*z*_,*g*_*i*_); the indirect link between *g*_*z*_ and *g*_*i*_, {(*g*_*z*_,*g*_*j*_), (*g*_*j*_,*g*_*i*_)},{(*g*_*z*_,*g*_*j*+1_),(*g*_*j*+1_,*g*_*i*_)},{(*g*_*z*_,*g*_*j*+2_),(*g*_*j*+2_,*g*_*j*+3_),(*g*_*j*+3_,*g*_*i*_)}. Mathematically, we calculate the IR score in the following steps.

First, a normalized adjacent matrix is generated by using the weighted average of neighbors, labeled as *U*. Given a gene *g*_*i*_ and *g*_*j*_, a normalize association score in *U* is calculated as follows.

1 $$ u_{ij} = \frac{e_{ij}}{\sum_{k\in V, (i.k) \in E}e_{ik}}  $$

Second, given a gene *g*_*z*_, its association with *g*_*i*_ is defined in terms of *g*_*j*_, we update the score iteratively. At each iteration *t*, the algorithm considers information from neighbors at path length=*t* (Eq. 2).

2 $$ r_{i}^{t+1} =\alpha o_{i} + (1-\alpha)u_{ij}r_{i}^{t}  $$

where *o*_*i*_ represents the original association score between *g*_*z*_ and *g*_*i*_, *α* is a weight parameter between 0 and 1. We can extend the Eq. 2 to calculate the iterative ranking-based association score for the whole network.

3 $$ R^{t+1}=\alpha O + (1-\alpha)U R^{t}  $$

where *O* is the adjacent matrix containing the original gene-gene relations in the input gene co-function network, *R* ^*t*^ and *R* ^*t*+1^ are adjacent matrices saving iterative gene association score in iterative *t* and *t*+1. The stopping criterion of the iterative process is defined as follows.

4 $$ \theta = \left\| R^{t+1}-R^{t}\right\|_{1} = \max_{j}\Sigma_{i=1}^{n}\left|(R^{t+1}-R^{t})_{i,j}\right|  $$

where *n* is the number of nodes involved in the network. The iteration stops until *θ* is smaller than a given threshold. The pseudo-code of the algorithm is shown in Algorithm 1.



### Step 2. calculating the similarity between two gene sets

Given two terms *t*_1_ and *t*_2_ in different GO categories *C*_1_ and *C*_2_, let *G*_1_ and *G*_2_ be gene set annotated by *t*_1_ and *t*_2_. Based on the global association score between genes calculated in last step, the association score of the two gene sets is calculated in this step. Given an adjacent matrix *R*, which includes the iterative ranking-based association scores between genes, the network-based similarity between *t*_1_ and *t*_2_ is defined based on their annotation sets as follows.

5 $$ Sim_{net}(t_{1},t_{2}) = \frac{|G_{1} \cup G_{2}| - |G_{1} - G_{2}| - |G_{2} - G_{1}|}{|G_{1} \cup G_{2}|}   $$

where *G*_1_ and *G*_2_ represent the gene sets annotated to *t*_1_ and *t*_2_ respectively, |*X*| is the number of genes in set *X*, *G*_1_∪*G*_2_ is union of set *G*_1_ and *G*_2_. Noted that we re-defined |*G*_1_−*G*_2_| in our method as follows:

6 $$ |G_{1} - G_{2}| = |G_{1}| - \sum\limits_{g_{i} \in G_{1}}\left(1 - \prod\limits_{g_{j} \in G_{2}}\left(1 - r_{ij}\right)\right)   $$

where *r*_*ij*_ is association score between genes *g*_*i*_ and *g*_*j*_ in network *R*. Particularly, if two gene sets *G*_1_ and *G*_2_ are identical, |*G*_1_−*G*_2_|=0. In summary, the term similarity *S* *i* *m*_*net*_(*t*_1_,*t*_2_) represents the association between *G*_1_ and *G*_2_ annotated by *t*_1_ and *t*_2_ based on the gene association in *R*.

### Step 3. calculating the cross-categories term similarity

In this step, we combine the network-based gene set similarities and the level information in GO to calculate the similarity between *t*_1_ and *t*_2_ in different categories. To overcome the “shallow annotation” problem, we take the level information of *t*_1_ and *t*_2_ in different categories into account.

7 $$ Sim_{GO} = \sqrt{\left(1-\frac{|G_{1}|}{|G_{C_{1}}|}\right)\cdot \left(1-\frac{|G_{2}|}{|G_{C_{2}}|}\right)}  $$

where $|G_{C_{1}}|$ and $|G_{C_{2}}|$ are the number of genes in the category *C*_1_ and *C*_2_. If *t*_*x*_ is close to the root of *C*_*x*_, $1-\frac {|G_{x}|}{|G_{C_{x}}|}$ is close to 0; if *t*_*x*_ is a specific term (far from the root), $1-\frac {|G_{x}|}{|G_{C_{x}}|}$ is close to 1. Equation (7) shows that the specific term pair are more likely to be identified.

Then, the similarity between *t*_1_ and *t*_2_ is calculated by integrating gene co-functional network, GO structure and gene annotations as:

8 $$ Sim(t_{1},t_{2}) = Sim_{net}\cdot Sim_{GO}  $$

Our previous work indicated that the relationships between two terms should be directed [21]. Therefore, we applied the term pair assignment method proposed in our previous work to look for the directions of the relationships. First, all similarities of term pairs across categories are computed with Eq. (8). Second, a user defined threshold is applied to filter term relationships with a threshold. Third, given a term *t*_1_ and a term set *T*_2_ that has connection to *t*_1_, the edge direction are deleted from *t*_1_ to *t*_2_ only if there is a term *t*_3_ satisfying that *t*_3_ is a descendant of *t*_2_ (*t*_2_,*t*_3_∈*T*_2_). In the end, we can get the directed relationships between terms in different GO categories.

## Results

In our experiment, we used BP and MF category as input to evaluate *C* *r* *o* *G* *O*2. To show the significance of *C* *r* *o* *G* *O*2, we compare *C* *r* *o* *G* *O*2 with *CroGO* [21], *ASR*-based [22] and *VSM*-based [23] methods. All the four methods are applied to a gold-standard set constructed with known pathway-to-reaction associations on yeast, which is also used as the evaluation data set in previous research [20, 21]. Then, we constructed a term association network for yeast between BP category and MF category.

The GO data and gene annotations were downloaded from GO official website in October 2015 [27]. We used yeastNet as the input co-function network, which contains 102,803 edges and 5483 genes [29]. *C* *r* *o* *G* *O*2 was implemented with java and JUNG library [30]. In the experiment, parameter *α* is set as 0.1. To determine the parameter *α*, we re-ran *C* *r* *o* *G* *O*2 by varying the parameter *α*. *C* *r* *o* *G* *O*2 achieve the best performance when *α*=0.1.

### Performance evaluation on gold-standard set

To test the performance of *C* *r* *o* *G* *O*2, we generated a “gold-standard” set based on the pathway-to-reaction interactions [20] in yeast. The process includes three parts: 1) a BP term is associated with a pathway based on GO biological process; 2) a metabolic pathway could be associate with several Enzyme Commission (EC) groups based on the enzymes catalysation; and 3) each EC can be linked to a MF term based on the association data from GO database [31–33]. Finally, the gold-standard set includes 334 MF-BP pairs. These 334 MF-BP term pairs are considered as the positive set. We also randomly selected 334 MF-BP term pairs as the random set. Note that similar gold-standard set generation method has been applied in previous research but on different data sources [20, 21]. Similarities of term pairs in both gold-standard set and random set are calculated using all four compared methods. We compared their performance based on receiver operating characteristic (ROC) curve [34] of each approach.

**Fig. 3:**
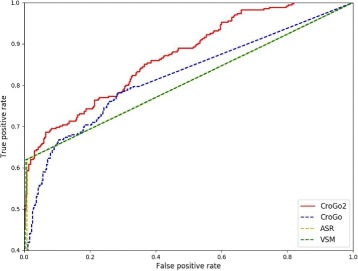
ROC curves for the four methods on the gold-standard sets of yeast. The red, blue, yellow and green lines represent CroGO2 (red), CroGO (blue), and ASR (yellow) and VSM (green) method respectively. Most portion of ROC curves of ASR and VSM are overlapping

The result showed clearly that *C* *r* *o* *G* *O*2 performs better than other three methods. Comparing the AUC score of the four methods showed that *C* *r* *o* *G* *O*2 had the highest AUC score (0.87) with the *CroGO* as the runner-up (Fig. 3). The AUC scores of *CroGO*, *ASR* and *VSM* are 0.82, 0.80 and 0.81 respectively. Table 1 shows that when the false positive threshold is 5%, the true positive rate of *C* *r* *o* *G* *O*2 is 66%, while the values of *CroGO*, *ASR* and *VSM* based approaches are 56, 59 and 59% respectively. *C* *r* *o* *G* *O*2 also has the highest true positive rate when the false positive rate is equal to 10 and 15%.

**Table 1:** The performance of ASR, VSM, CroGO and CroGO2 measures on yeast gold-standard set

Organism	Measure	TP rate (when	TP rate (when	TP rate (when
		FP rate =5%)	FP rate =10%)	FP rate =15%)
*Yeast	ASR	59%	/	/
	VSM	59%	/	/
	CroGO	56%	65%	67%
	CroGO2	66%	69%	71%

In summary, the evaluation test indicates that *C* *r* *o* *G* *O*2 has produced better performance than the other measures.

### Robustness test of *CroGO*2

*C* *r* *o* *G* *O*2 combined the co-function network. To test whether varied the co-function network density would affect the performance of *C* *r* *o* *G* *O*2, we randomly deleted 50% of edges in the co-function network and used the low-density co-function network as input.

**Fig. 4:**
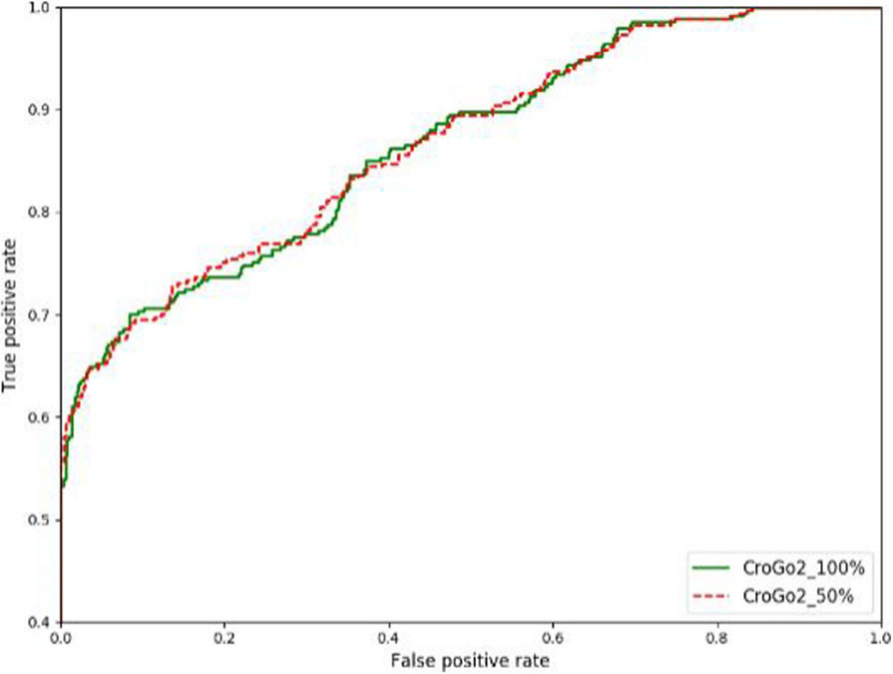
ROC curves for the robustness test of *C* *r* *o* *G* *O*2 with different co-function network densities

The result shows that there was no significant different between results using two networks with different densities (Fig. 4). The AUC scores using the full network and low-density network are 0.870 and 0.869, which are almost the same.In summary, the experiment result shows that *C* *r* *o* *G* *O*2 has high robustness.

## Discussion

In this section, we linked BP and MF terms to generate a term association network for yeast. The cross-category term association network can provide a convenient way for researchers to use *C* *r* *o* *G* *O*2.

A reliable MF-BP association network is generated by calculating pairwise similarities of all MF and BP terms and applying a strict FDR threshold (in this case we use *F* *D* *R*<0.05). Finally, the association network includes 1406 MF terms, 2305 BP terms, and 8531 linkages.

**Fig. 5:**
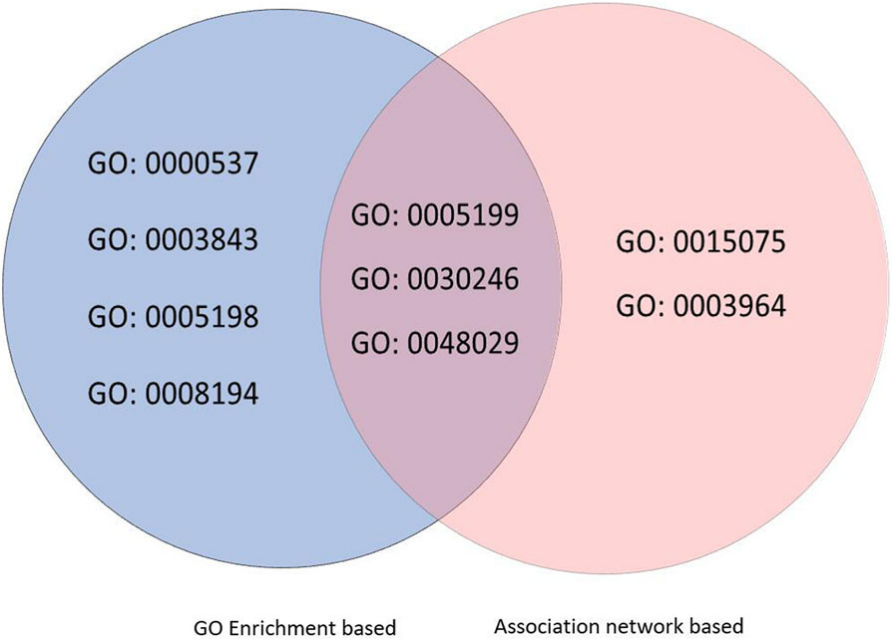
Venn diagram of *T*_*MF*_ and *T* *MF*′. *T*_*MF*_ is the set of enriched MF terms. *T* *MF*′ is the set of MF terms associated with the enriched BP terms

To show the power of the MF-BP association network *N*, we test whether the result based on association network has an agreement with the result based on GO enrichment. Given a set of genes *S* with particular function, we can get its enrichment results based on BP category and MF category separately. The enriched term sets of *S* on BP and MF category are labeled as *T*_*BP*_ and *T*_*MF*_ respectively. Given *T*_*BP*_ and *N*, we can find out the MF terms, saved as *T* *MF*′, connect with terms in *T*_*BP*_ based on *N*. We can check whether overlap terms can be identified between *T*_*MF*_ and *T* *MF*′. For example, we find a set of genes which are associated with the phenotype “adhesion” from the yeast phenotype ontology [35]. The gene set is {*C* *D* *C*33,*C* *I* *S*3,*C* *W* *P*2,*F* *I* *G*2,*F* *K* *S*3,*F* *L* *O*10,*F* *L* *O*11,*F* *L* *O*5,*F* *L* *O*9,*P* *I* *R*3,*S* *C* *W*4}. Following the aforementioned experiment protocol, the result is shown in Fig. 5. It is shown that three terms (GO:0005199, GO:0030246 and GO:0048029) can be identified by both GO enriched-based and MF-BP association network-based methods.

Furthermore, the top 20 term associations, which do not have identical annotation set, are shown in Table 2. We found biological evidence from literature or term definition for 15 of them. The rest 5 new conceptual connections may be new knowledge not found in previous study.

**Table 2:** Top 20 term associations that were identified by *C* *r* *o* *G* *O*2

BP Name	MF Name	Evidence
butanediol biosynthetic process	(R,R)-butanediol dehydrogenase activity	New
glutamine biosynthetic process	glutamate-ammonia ligase activity	[36]
putrescine biosynthetic process	ornithine decarboxylase activity	[37, 38]
acetyl-CoA biosynthetic process from acetate	acetate-CoA ligase activity	New
alanine catabolic process	L-alanine:2-oxoglutarate aminotransferase activity	[39]
siroheme biosynthetic process	precorrin-2 dehydrogenase activity	[40]
trehalose catabolic process	alpha,alpha-trehalase activity	[41]
asparagine catabolic process	asparaginase activity	[42]
lysine biosynthetic process	aromatic-amino-acid:2-oxoglutarate aminotransferase activity	[43, 44]
glycerol biosynthetic process	glycerol-1-phosphatase activity	New
threonine catabolic process	L-threonine ammonia-lyase activity	New
peptide alpha-N-acetyltransferase activity	N-terminal protein amino acid acetylation	[45]
glutathione catabolic process	gamma-glutamyltransferase activity	[46]
alanine biosynthetic process	L-alanine:2-oxoglutarate aminotransferase activity	[47]
positive regulation of histone H3-K36 methylation	TFIIF-class binding TF activity	New
siroheme biosynthetic process	uroporphyrin-III C-methyltransferase activity	[48]
siroheme biosynthetic process	sirohydrochlorin ferrochelatase activity	[40]
glutathione biosynthetic process	glutamate-cysteine ligase activity	[49, 50]
positive regulation of telomere maintenance via telomerase	Hsp90 protein binding	[51, 52]
chorismate biosynthetic process	3-deoxy-7-phosphoheptulonate synthase activity	[53]

## Conclusions

Identifying the relationships between GO terms in different categories is vital for understanding the biological mechanism and inferring gene function. Recently, researchers have begun to employ gene co-function networks to calculate the similarity between terms in different GO categories. In this article, we proposed a novel approach, called *C* *r* *o* *G* *O*2, to measure the cross-categories GO term similarities by incorporating level information in gene ontology with both direct and indirect interactions in the gene co-function network. *C* *r* *o* *G* *O*2 has the following advantages: 1) CroGO2 performs better than existing methods by taking the global interactions in the gene co-functional network into account; 2) A novel iterative ranking-based method is developed to measure the relationship between two gene sets; 3) A cross-categories term association network was constructed by selecting the high-quality associations. To demonstrate the advantages of *C* *r* *o* *G* *O*2, we compare it with three existing approaches *CroGO*, *ASR* and *VSM*. The experiment on a gold standard set shows that *C* *r* *o* *G* *O*2 performs better than other methods. Furthermore, *C* *r* *o* *G* *O*2 has the high robustness to the co-function network density. We also generated a genome-specific term association network of yeast. The linkages in the association network can be supported by literature. Given a gene set, the related terms identified by using the association network have overlap with the related terms identified by GO enrichment analysis.
